# Recent Advances and Next Breakthrough in Immunotherapy for Cancer Treatment

**DOI:** 10.1155/2022/8052212

**Published:** 2022-03-18

**Authors:** Ling Yang, Qian Ning, Sheng-song Tang

**Affiliations:** ^1^Hunan Provincial Key Laboratory of Tumor Microenvironment Responsive Drug Research, and Institute of Pharmacy & Pharmacology, University of South China, Hengyang 421001, China; ^2^Hunan Province Key Laboratory for Antibody-Based Drug and Intelligent Delivery System, School of Pharmaceutical Sciences, Hunan University of Medicine, Huaihua 418000, China; ^3^Department of Pharmacy, The First People's Hospital of Huaihua City, Huaihua 418000, China; ^4^College of Bioscience and Biotechnology, Hunan Agricultural University, Changsha 410000, China

## Abstract

With the huge therapeutic potential, cancer immunotherapy is expected to become the mainstream of cancer treatment. In the current field of cancer immunotherapy, there are mainly five types. Immune checkpoint blockade therapy is one of the most promising directions. Adoptive cell therapy is an important component of cancer immunotherapy. The therapy with the cancer vaccine is promising cancer immunotherapy capable of cancer prevention. Cytokine therapy is one of the pillars of cancer immunotherapy. Oncolytic immunotherapy is a promising novel component of cancer immunotherapy, which with significantly lower incidence of serious adverse reactions. The recent positive results of many clinical trials with cancer immunotherapy may herald good clinical prospects. But there are still many challenges in the broad implementation of immunotherapy. Such as the immunotherapy cannot act on all tumors, and it has serious adverse effects including but not limited to nonspecific and autoimmunity inflammation. Here, we center on recent progress made within the last 5 years in cancer immunotherapy. And we discuss the theoretical background, as well as the opportunities and challenges of cancer immunotherapy.

## 1. Introduction

**Figure 1:**
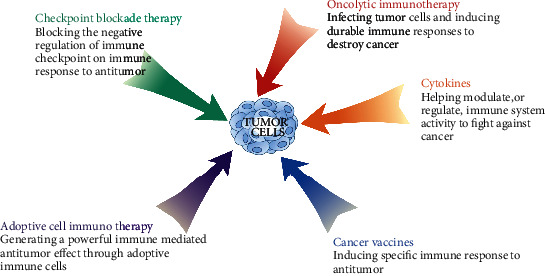
Components and brief mechanisms of cancer immunotherapy.

Cancer is a devastating disease, which has been one of the main threats to human health. Worldwide, nearly 10 million people will die from cancer in 2020 [1]. Surgery, radiotherapy, and chemotherapy are the three traditional treatments of cancer, but these methods have certain limitations, such as traumatic, low targeting, serious toxicity, and drug resistance [2]. Furthermore, they often fail to provide long-term survival benefits for patients with advanced solid tumors, according to clinical practice. Along with the deepening research of tumor immunology, cell biology, and molecular technology, scientists find that the tumor microenvironment (TME) is immunosuppressive. Studies have shown that cancer development and metastasis are highly positively correlated with immunosuppression [3]. Cancer immunotherapy, which harnesses the body's immune system to eradicate tumor cells, is widely researched. The components and brief mechanisms of cancer immunotherapy are shown in Figure 1. Thousands of clinical trials have proved that cancer immunotherapy is becoming a powerful new approach to cancer treatment. Despite the promising prospects, the clinical application of immunotherapy still faces some challenges in terms of effectiveness and safety. In this review, the five cancer immunotherapies mentioned above are overviewed, and their clinical status, advantages, and disadvantages are discussed.

## 2. Immune Checkpoint Blockade Therapy

Immune checkpoints refer to immunosuppressive molecules. Physiologically, immune checkpoints are important for maintaining immune tolerance regulating immune responses and preventing tissue damage. Nonetheless, the high expression of checkpoints can mediate tumor immune evasion by inhibiting immune cell function, in the development and activation of tumors [4]. Fortunately, immune checkpoint inhibitors can block the transmission of immunosuppressive signals, then restore or enhance the body's antitumor immune response.

**Figure 2:**
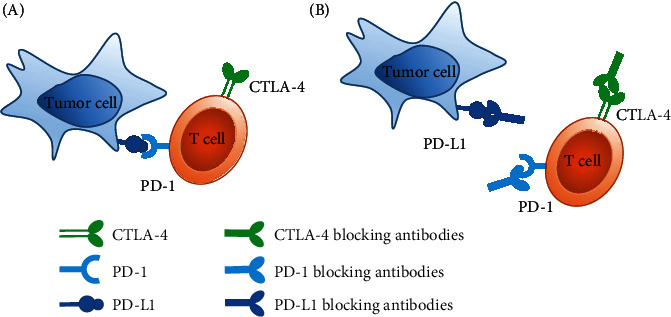
The brief antitumor mechanism of CTLA-4 and PD-1/PD-L1 blocking antibodies. (a) In the tumor microenvironment, the T cell surface is highly inhibited by inhibitory immunoregulatory receptors, such as CTLA-4 and PD-1/PD-L1, which prevents the immune activation of T cells and the killing of tumors. (b) The use of PD-1/PD-L1 or CTLA-4 blocking antibodies can eliminate the immunosuppressive effects of PD-1/PD-L1 or CTLA-4, thereby activating the immune response of T cells to kill tumors.

The main representatives of immune checkpoints are cytotoxic T lymphocyte antigen 4 (CTLA-4), programmed cell death protein1 (PD-1), and programmed cell death ligand1 (PD-L1). CTLA-4 is expressed on the activated CD^8+^ and CD^4+^ T cells. During the early activation of T cells, CTLA-4 competes with the costimulatory receptor CD28 to bind to ligands B7-1 and B7-2 expressed on the antigen-presenting cells (APCs). Then inducing the downstream negative regulation of immune response, which leads to the suppression of T cell proliferation and the IL-2 secretion [5, 6]. It ultimately inhibits the adequate immune response to tumor cells. Like the CTLA-4, PD-1 is also a transmembrane protein expressed on T cells. PD-L1 is one of its ligands, which can be expressed on tumor cells, APCs, and T cells themselves. When PD-1 binds to PD-L1, it reduces the response of T cells to T cell receptor (TCR) stimulation signals through PI3K-AKT and JAK-STAT signaling pathways [7, 8]. Leading to the suppressive antitumor T cell responses, the brief antitumor mechanism of CTLA-4 and PD-1/PD-L1 blocking antibodies is shown in Figure 2.

Thus far, one anti-CTLA-4 antibody (ipilimumab), three anti-PD-1 antibodies (pembrolizumab, nivolumab, and cemiplimab), and three anti-PD-L1 antibodies (atezolizumab, avelumab, and durvalumab) for the treatment of different malignancies have been approved by the United States Food and Drug Administration (FDA) [9]. Ipilimumab, a monoclonal antibody directed against CTLA-4, was approved by the FDA for patients with metastatic melanoma in 2011 [10]. It is the first clinically approved immune checkpoint inhibitor. When ipilimumab was used to treat metastatic melanoma, 20% of patients survived more than 4 years, and a small percentage of patients survived for 10 years or more [11]. Ipilimumab is also widely used in the treatment of tumors such as lung cancer, kidney cancer, and prostate cancer [12]. But the effectiveness is less than metastatic melanoma. Efficiencies below 10% are usually unsatisfactory. Generally speaking, the antitumor treatment efficiency of PD-1/PD-L1 blockers is better than CTLA-4 blockers. In patients with advanced melanoma, pembrolizumab was found to be more effective than ipilimumab at extending progression-free survival and overall survival [13]. Nivolumab, for the patients with classic Hodgkin's lymphoma, the treatment response rate is more than 80% [14]. And for many patients with cancer, the curative effect of PD-1/PD-L1 blocking antibody is above 10%. In addition to the antitumor efficacy, the adverse effects cannot be ignored. The most common are immune-related adverse events (irAEs), such as rash, colitis, diarrhea, hepatotoxicity, and endocrinopathies. There are even occasional fatal adverse events [15]. The clinical promotion of immune checkpoint inhibitors is limited by relatively low response rates and relatively high treatment-related toxicity. Some biomarkers can predict the therapeutic effects and the adverse reactions of immune checkpoint blockade therapy. It brings hope to the widespread application of immune checkpoint blocking therapy. Higher mutational load and neoantigen loads are significantly associated with the clinical benefit of anti-CTLA-4 antibodies [16]. Tumor PD-L1 expression was significantly associated with the overall response rate (ORR) of PD-1/PD-L1 inhibitors. The ORR of the PD-L1 positive group was 34.1% while the PD-L1 negative group was 19.9% [17]. These biomarkers will be helpful in the identification of cancer patients who can benefit from the immune checkpoint blockade therapy. Meanwhile, the increase in expression of CD177 and CEACAM1 is closely related to colitis after ipilimumab treatment [18]. That is to say, for patients treated with ipilimumab, CD177 and CEACAM1 can help to take countermeasures in advance to reduce the damage caused by immune-related colitis. In addition, many potential biomarkers are useful to predict the side effects and therapeutic efficacy; for instance, mismatch repair (MMR) deficiency, interferon-*γ* (IFN-*γ*) related mRNA profile, and T-cell invigoration to tumor burden ratio [19–21]. When the predictive effect of biomarkers is verified in a larger patient cohort, precision medicine, and immune checkpoint blockade therapy will take a new step.

## 3. Adoptive Cell Therapy

**Figure 3:**
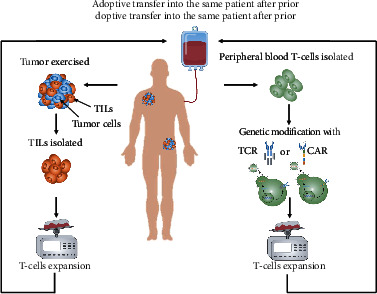
The mechanism of adoptive cell therapy. ACT with TILs separates TIL cells from tissues near the tumor, and large-scale expand in vitro, then reintroduce into the patient. ACT with TCR or CAR separates T cells of patient peripheral blood and is genetically modified to express TCR or CAR, then it can specifically recognize and attack tumor cells when reinfused into the patient. Before the adoptive transfer to the patients, they are treated with a lymphodepletion adjustment regimen.

Adoptive cell therapy (ACT) extracts immune-competent cells from cancer patients, genetically modifies or massively expands immunecompetent cells in vitro to increase immune activity, and then reinjects them into cancer patients to enhance the body's anti-tumor immune function [22, 23]. Immunocompetent cells include lymphokine-activated killer cells (LAK), chimeric antigen receptor-modified T (CAR-T) cells, chimeric antigen receptor-modified nature killer cells (CAR-NK), T cell receptor-modified T (TCR-T) cells, cytokine activation killing (CIK) cells, and tumor-infiltrating lymphocytes (TILs). The use of TILs, TCR-T cells, and CAR-T cells is the most studied of all ACT therapies. And the mechanisms of them are shown in Figure 3.

Rosenberg et al. first reported that TILs could promote tumor regression in patients with metastatic melanoma [24]. In metastatic melanoma patients receiving ACT with TILs, the objective response rate is 40-50%, including 10-20% complete tumor regression has been reported many times [25–27]. Whereas amplifiable antitumor TILs are only found in a few types of tumors, and the expansion process of TILs is complex [28]. The successful use of CD137 to separate and select TILs gives us a good idea for enriching tumor-reactive TILs [29]. The key to the ACT with TILs is to improve the quality and characterization of T cells, and how to simplify the method for obtaining tumor-specific T cells will be the focus of the next research.

The genome-editing method that won the 2020 Nobel Prize in Chemistry also promotes the development of adoptive cell therapy. Being frequently exposed to foreign nucleic acids, bacteria and archaea have developed an ingenious adaptive defense system, called CRISPR-Cas [30]. ACT with genetically modified T cells can recognize tumor antigens through related tumor-reactive TCR or CAR. A study of 20 patients with advanced myeloma confirmed that NY-ESO-1-specific TCR-T cells mediate sustained antigen-specific antitumor effects [31]. In a pilot trial, after NY-ESO-1 treatment, the 3-year and 5-year overall survival rates of patients with synovial cell sarcoma were estimated to be 38% and 14%, respectively, while the corresponding estimated survival rates for melanoma patients were 33%. But two patients developed cardiogenic shock after an infusion of TCR-T cells and died within a few days [32]. ACT with TCR-T shows some efficacy against the tumor. But it has certain individual differences, which not only lead to differences in antitumor efficacy but may even threaten the lives of patients. In order to reduce toxicity, it is extremely beneficial to identify personalized targetable antigens that can be expressed on tumors but not on healthy tissues. Neoantigens due to tumor-specific mutations will surely become a hot spot for the future. Besides, MHC class I complexes are usually downregulated in cancer cells [33]. TILs and TCR modified T cells recognize antigen in an MHC-dependent manner also limit their clinical application. The antigen recognition ability of CAR-T cells is based on tumor surface proteins and can eliminate the limitations of the MHC to present antigens.

ACT with CAR-T cells makes use of transgenic technology to express CAR in T cells, which usually consists of an extracellular antigen binding moiety (such as antibody scFv) fused to an intracellular signaling domain for T cell activation [34, 35]. The transformation of the signal domain in the CAR structure enables CAR to play a variety of T cell functions, such as amplification, cytokine secretion, and tissue selectivity [36, 37]. It can further expand the scope and efficacy of ACT. Extracellular glycoprotein CD19 is the most common cellular target for ACT with CAR-T cells, which is a marker of B cell tumors. CD19 CAR-T has been successfully used to treat patients with refractory chronic lymphoma. The efficacy of the three patients was more than half a year, and 2 of them were completely relieved [38]. The CAR-T therapies (tisagenlecleucel and axicabtagene ciloleucel) for adult and pediatric B cell malignancies are approved by the FDA in 2017 [39, 40]. Besides, trials of CAR-T treatment targeting CD22 [41] and CD30 [42] are also underway. And the new cellular targets for ACT with CAR-T cells bring new hope for reversing relapse. CAR-T cell therapy is not only a promising therapy to overcome hematological tumors but also a method with great therapeutic potential in solid tumors. Although previous studies have shown poor results and varying degrees of toxicity. A recent CAR-T cell targeting IL-13R*α*2 has induced complete regression of metastatic glioblastoma in patients [43]. But the adverse effects like cytokine release syndrome (CRS) and on-target, off-tumor toxicity cannot be ignored [40, 44]. In an attempt to reduce the toxicity, many methods are researched, such as enhancing the targeting of T cells to tumor-specific antigens, inhibiting the immunity nonspecifically, and developing novel drug delivery systems [45, 46]. But the overall effect is not very satisfactory, and further researches are necessary.

## 4. Cytokines

Cytokines are polypeptides, proteins, or glycoproteins with a molecular weight usually under 30 kDa, which are involved in transmitting the signals of cell proliferation, differentiation, inflammation, or anti-inflammatory [47, 48]. The main feature of cytokine therapy is that it can directly promote the growth and immune activity of immune cells.

Interleukin-2 (IL-2) is critical to the activation, growth, and survival of T cells and NK cells and maintains the delicate balance between autoimmunity and antineoplasm 3 surveillance. It has been proved that the high-dose IL-2 (HDIL-2) has the superior antitumor effect while the low doses of IL-2 can induce the proliferation of suppressor T cells (Tregs), which suppress the activation of the immune system, weakening the antitumor efficacy. The FDA approved HDIL-2 for the therapy of metastatic renal cell carcinoma (RCC) in 1992 and the therapy of metastatic melanoma in 1998 [49, 50]. Nevertheless, the high dose of IL-2 can cause the capillary leak syndrome, which is featured by multiorgan damage, such as hypotension, lung edema, and renal failure resulting from extravasation of fluid into the organs. According to the latest research, FSD13, produced by a selective amino acid replacement, can selectively inhibit the IL-2 mediated Treg proliferation and increase the IL-2 function of stimulating the antitumor immune cell [51]. It provides a new method to reduce systemic toxicity and improve the efficacy of IL-2-based immunotherapy.

Interferon-alpha (IFN-*α*) is another representative cytokine that can play an important role in the body's immune regulation and antitumor immune response. IFN-*α* not only activates immune cells directly but also effectively activates the systemic immune response by reversing the immunosuppression of effector mesenchymal stromal cells (MSCs) [52]. IFN-*α* was approved in 1986 for the therapy of hairy cell leukemia and in 1995 for the therapy of melanoma [53, 54]. However, the use of IFN-*α* can be quite toxic, especially at high doses. Elevated liver enzymes, neutropenia, thrombocytopenia, and leukopenia are common toxicities. Besides, many other cytokines like interleukin-8 (IL-8), interleukin-10 (IL-10), interleukin-12 (IL-12), interleukin-15 (IL-15), interleukin-21 (IL-21), granulocyte-macrophage colony-stimulating factor (GM-CSF), transforming growth factor-beta (TGF-*β*), and tumor necrosis factor-alpha (TNF-*α*) are underinvestigated.

Of note, the short half-life, the relatively low objective response rates, and high toxicity associated with high doses of some cytokines administration have seriously limited the widespread research and clinical application of cytokines [55, 56]. To break the limitations, many methods are taken. Cytokines injection into tumor lesions was tried, and cytokine fusion proteins were engineered to enhance the antitumor effect [57–59].

## 5. Cancer Vaccines

Cancer vaccine takes advantage of tumor-associated antigens (TAAs) or tumor-specific antigens (TSAs) to stimulate the immune system, especially a robust and long-lasting immune response of CD^8+^ T cells to inhibit the growth, metastasis, and recurrence of tumor cells [60]. There are numerous different platforms of the cancer vaccine, mainly including cell-based, RNA-based, DNA-based, and protein/peptide-based preparations [61]. According to the clinical use of cancer vaccines, they are divided into two categories: preventive and therapeutic. The preventive cancer vaccine aims to prevent tumor occurrence by inducing immune response while the therapeutic cancer vaccines are designed to eradicate tumor cells by inducing or enhancing the tumor-specific immunoreactions [62]. Currently, the relatively successful preventive cancer vaccines are cervical cancer vaccines by targeting human papillomavirus (HPV). The approved cervical vaccines Gardasil and Cervarix resulted in the reduction of the prevalence of cervical intraneoplasia in young women with the administration of the vaccine during adolescence [63, 64]. However, they are mostly invalid once suffering from cancer or chronic infection with HPV. Therefore, not only preventive cancer vaccines but also therapeutic cancer vaccines are needed. It is inspiring that sipuleucel-T (Provenge) can extend the overall viability of patients with hormone-resistant prostate cancer by an average of 3 months [65]. And it is approved by the FDA for metastatic castration-resistant prostate cancer. Although after using cancer vaccines, many experiments have shown that some patients with advanced cancer have good clinical effects, the partial remission (PR) or complete remission (CR) rate is basically below 10%, or even below 5%. So far, in phase 3 randomized trials, there is no other therapeutic cancer vaccine has yet shown noteworthy clinical efficacy except the sipuleucel-T [66–69].

The efficacy of the cancer vaccine is primarily dependent on immunogenicity, host immunosuppression, preferential expression of tumor antigen, and the delivery of cancer vaccine [70]. The tumor-specific antigens, also known as neoantigens, are ideal cancer vaccine targets, due to the highly immunogenic, lower risk of self-tolerance, less common tumor antigen deletions, and lower risk of autoimmune reactions [71, 72]. And next-generation sequencing (NGS) brings a technological breakthrough to the screening of neoantigens. In a study of 13 patients with melanoma who had no clinically visible residual surgery after surgery, 8 patients had complete tumor disappearance and no recurrence after receiving the personalized RNA mutanome vaccine [73]. In 6 melanoma patients who had received personal neoantigen vaccines, 4 patients had complete tumor disappearance, no recurrence, and 2 patients had tumor recurrence but were completely relieved after treatment with the programmed death receptor 1 (PD1) monoclonal antibody (pembrolizumab) [74]. Based on these studies, individualized cancer vaccines have strong theoretical support, and new approaches to individualized anticancer immunotherapy have been opened up. However, the preparation process of personalized cancer vaccines is complicated and the preparation time is long, which needs to be further explored and solved. In detail, rapid screening of highly immunogenic neoantigens, selection of appropriate immune adjuvants, rapid verification of vaccine potency, and shortening of preparation time will be the focus of future cancer personalized vaccine research.

## 6. Oncolytic Immunotherapy

Oncolytic immunotherapy is based on oncolytic viruses (OVs). OVs can lead to lysis of the tumor cells and the activation of the innate and adaptive immune response, by specifically replicating in cancer cells without damaging normal cells [75, 76]. That is to say, oncolytic immunotherapy can play the antitumor effect by directly lysing tumors and stimulating the body's immunity. It fights malignancies without dependence on specific antigen expression, which makes it superior to other immunotherapy approaches. In 2015, a genetically engineered oncolytic herpes simplex virus (HSV), talimogene laherparepvec (T-VEC), was approved for the treatment of advanced melanoma by the United States FDA [77, 78].

However, the immune response activated by OVs is a double-edged sword, because it consists of the antitumor effect and the viral immune response [79]. On the one hand, OVs stimulate the immune system to recognize cancer cells and activate antitumor immunity mainly by inducing immunogenic cell death (ICD) of them [80–82]. On the other hand, OVs are identified by the immune system as causative agents. The immune system generates the antiviral response, which could diminish the efficacy of the antitumor by clearing the virus prematurely [79, 83]. The intratumoral injection is adopted for its safety in oncolytic immunotherapy. It can prevent the humoral immunity from removing viruses and repeated intratumoral injection can induce a strong immune response against the tumor [84]. Whereas the majority of cancers are metastatic, intravenous injection is an ideal method of delivery for the disseminated tumors [85, 86]. How to avoid the early elimination of OVs caused by antiviral response becomes especially important. Moreover, we cannot simply remove the antiviral response, because it can help to reverse the tumor-mediated suppression of immunity [87]. Perfect oncolytic immunotherapy requires a desirable balance of immune responses between antitumor and antiviral. Numerous approaches have been studied to obtain the above balance and the greatest therapeutic effect, such as way like genetic modification [88–90], depleting antibodies [91], polymer coating [92], and carrier cells [93].

## 7. Conclusion and Prospects

The immune system can specifically attack cancer cells, coupled with its ability to adapt to progressive tumors, and its long-lasting memory function, making cancer immunotherapy the most promising therapy for durable control of cancer. Remarkable success has been achieved in cancer immunotherapy. Several cancer immunotherapy agents have been approved for the treatment of many types of malignancies with durable and impressive clinical responses. But the toxic side effects, insufficient immune responses, and the immunosuppressive environment of tumors create great challenges to therapeutic efficiency. Our group has long been committed to the research and development of antitumor drugs such as immune evasion mechanism of tumor cells [94], screening, and delivery of antitumor drugs [95]. And developing a targeted drug delivery system to improve the efficiency of the cancer immunotherapy agents in the next direction of our efforts.

In addition, there are many promising research directions, such as screening more suitable immunotherapy targets to reduce toxic effects, finding reliable biomarkers to guide the specific immunotherapy of cancer, and combining immunotherapy with other appropriate cancer therapies to expand the scope of application. Clinical trials of cancer immunotherapy continue to expand the indications for these therapies and explore new ways to harness the immune system to treat cancer. The prospects of cancer immunotherapy research are immeasurable, and its extensive development and rapid advancement bring new dawn and hope to cure cancer.
